# Critical skills: qualitative content analysis of medical residents in social skills training with RPGs

**DOI:** 10.3389/fpsyg.2026.1691145

**Published:** 2026-03-20

**Authors:** Victor Henrique Oyamada Otani, Rafael Condoretti Novaes, Julia Pedron, Pedro Chen Nabhan, Thaísa Malbar Rodrigues, Ryo Chiba, Henrique do Amaral Duarte, João Vitor Guedes, Felipe Oliveira de Aguiar, Lucas Murrins Marques, João Ricardo Nickenig Vissoci

**Affiliations:** 1 Mental Health Department, Santa Casa de São Paulo School of Medical Sciences, São Paulo, Brazil; 2 Artificial Intelligence Research Division, Infinity Doctor's Inc., São Paulo, Brazil; 3 Independent Researcher, São Paulo, Brazil; 4 Division of Translational Health Sciences, Department of Emergency Medicine, Duke Global Health Institute, Duke University, Durham, NC, United States

**Keywords:** cognitive behavioraltherapy, consolidated framework for implementation research, gamification, role playing games, social skills training

## Abstract

The present study aimed to assess the viability of Social Skills Training through RPGs during the SARS-CoV-2 pandemic, employing qualitative discourse analysis of residents. Focus groups were conducted with a convenience sample of psychiatry residents who volunteered to participate in the RPG-based training. Eleven residents took part in video conference focus groups, with one researcher acting as a non-participant observer. Over a period of 4 months, residents engaged in weekly 3-h sessions structured around five fundamental steps: rapport building, task discussions, goal setting, gaming, and feedback. Results indicate positive impacts on group dynamics, communication skills, cooperation, assertiveness, empathy, mental health, and quality of life. However, given the exploratory nature of this study, further research incorporating pre- and post-intervention assessments is warranted to thoroughly evaluate the intervention’s effectiveness.

## Introduction

Medical residency is widely recognized as a period of intense psychological and occupational demands, including sleep deprivation, frequent exposure to patient suffering, restricted autonomy, and high clinical workload. These conditions create a highly stressful environment and are associated with increased rates of depressive symptoms, anxiety, burnout, and other adverse outcomes when compared to the general population (Shapiro et al., 2000; Sen et al., 2010; Pereira-Lima et al., 2016; Pereira-Lima et al., 2019). Additional consequences include substance use, suicidal ideation, medical errors, and reduced adherence to safety protocols (Fahrenkopf et al., 2008; West, 2009; de Oliveira et al., 2013). The COVID-19 pandemic further intensified these stressors, exacerbating depression, anxiety, and social isolation among residents (Brooks et al., 2020).

Evidence suggests that a stronger repertoire of social skills—such as communication, empathy, assertiveness, and teamwork, serves as a protective factor against stress-related disorders and burnout during residency (Pereira-Lima et al., 2016; Furtado et al., 2003). Consequently, Social Skills Training (SST) has been proposed as an important preventive strategy in medical education (Oliveira Bortolatto et al., 2022; Addington et al., 2021). SST aims to enhance residents’ interpersonal competencies, thereby mitigating the psychological burden associated with clinical training.

In previous work, our group developed a Social Skills Training protocol using tabletop role-playing games (ttRPGs) grounded in Cognitive Behavioral Therapy principles (Otani et al., 2024). Emerging evidence suggests that ttRPG-based interventions may reduce social anxiety, enhance social skills, and increase confidence in real-world interactions (Varrette et al., 2023; Abbott et al., 2022). However, such interventions have not yet been evaluated within medical residency training.

Simulation-based learning is well established in medical education, particularly for developing technical and procedural competencies (Dawson et al., 2007; Thompson Bastin et al., 2017). Nevertheless, its application to social skills development remains limited, and traditional simulation approaches may face barriers such as high costs, limited generalization, and participant stigma (Cardoso et al., 2023).

Role-playing games (RPGs) represent a distinct form of structured social simulation. Unlike conventional simulations, RPGs rely on collaborative storytelling, negotiation, and shared decision-making within fictional yet socially meaningful scenarios. Research in educational and healthcare contexts indicates that RPG participation may enhance empathy, emotional regulation, reflexivity, and prosocial behavior (Rivers et al., 2015; Abbott et al., 2022; Clarke et al., 2019). RPGs can be implemented online or in person and require minimal material resources, making them adaptable to diverse educational environments.

These characteristics suggest that RPG-based interventions may offer a structured yet flexible platform for developing social competencies and fostering psychological well-being, not as formal psychiatric treatments, but as guided spaces for experiential learning and reflective practice. This potential is particularly relevant for medical residents, whose professional roles demand continuous interpersonal negotiation under emotionally demanding conditions.

Despite this promise, no studies to date have evaluated RPG-based Social Skills Training specifically among medical residents. Therefore, the objective of this study was to assess the feasibility of Social Skills Training through RPGs using qualitative discourse analysis of residents’ experiences. The findings aim to inform the development of structured, theory-based interventions for medical training contexts.

## Method

This study employed a qualitative design and adhered to the Consolidated Criteria for Reporting Qualitative Research (COREQ) guidelines (Tong et al., 2007), considered a robust framework for healthcare qualitative research.

### Researchers

The focus groups were facilitated by Dr. JRNV (psychologist and researcher), who also served as the game facilitator (Dungeon Master), and Dr. VHOO (psychiatrist at the ISCMSP Psychiatry Institute), who acted as co-facilitator. Both have extensive experience in qualitative research and focus group methodology (Salci et al., 2022; El-Gabri et al., 2020).

Individual interviews were conducted by Me. RAC and Me. JVCG (both psychologists), who also provided logistical support during the intervention sessions. As facilitator, Dr. JRNV was responsible for designing and conducting the narrative scenarios based on the Dungeons & Dragons system, introducing structured social dilemmas aligned with targeted interpersonal competencies. The facilitator moderated narrative developments, portrayed non-player characters, and guided interaction dynamics throughout the sessions.

All researchers had prior experience with role-playing games. This familiarity supported technical management of the intervention format but did not involve pre-existing therapeutic relationships with participants.

### Participants

Participants comprised a convenience sample of 11 psychiatry residents from the Santa Casa de São Paulo School of Medical Sciences (ISCMSP). Residents were invited to participate voluntarily in the Social Skills Training via Role-Playing Games project, entitled “Critical Skills.”

Recruitment occurred between March and September 2021, following ethical approval obtained in January 2021. During the first week of residency, supervising physicians introduced the project and informed residents that the intervention aimed to develop empathy, communication, leadership, cooperation, and interpersonal connection skills. A subsequent videoconference provided detailed explanations regarding objectives, procedures, study phases, and ethical safeguards, after which informed consent was obtained.

Although participants were informed about the general objectives related to social skills development, they were not briefed on the specific narrative structure, sequencing of challenges, or pedagogical framework of the sessions. This approach ensured informed and voluntary participation while minimizing priming effects that could influence spontaneous in-game behavior (Table 1).

**Table 1:** Individual experiences of the medical residents during the isolation.

Category/Theme	Codes/Subthemes
Residency experiences	Stress situations on the residency
	Reinforcing experiences on the residency
Emotional and behavioral difficulties	Difficulties in social situations
	Anxiety symptoms
	Difficulties with playful repertoire
	Difficulties in generalizing behaviors learned from playing RPG
Internet use to build friendship	Presence of previous experiences with virtual friendships
	Absence of previous experiences with virtual friendships
Motivation for playing RPG	There was previous interest in playing RPG
	Possibility of scientific research as a motivational factor to play RPG
	Pandemic as a motivational factor to join the RPG group

Residents were free to decline or withdraw at any time without academic or professional consequences. No participants withdrew from the study. All participants were over 18 years old and provided written informed consent.

### Study design

The study adopted a phenomenological approach to explore how medical residents experienced the ttRPG-based intervention and how these experiences related to their social skills development and broader personal domains. To ensure theoretical coherence, the intervention was grounded in principles of Cognitive-Behavioral Therapy (CBT)—particularly behavioral reappraisal, emotional identification, and perspective-taking—which align naturally with the interactive and reflective dynamics of role-playing activities.

Beyond its narrative structure, the ttRPG format was intentionally designed as a Social Skills Training (SST) environment, informed by Otani et al. (2024) and primarily based on the theoretical-practical model of Del Prette and Del Prette (2019). In this framework, social skills are organized into essential behavioral classes—such as communication, empathy, assertiveness, conflict management, group coordination, and public speaking—that directly correspond to the interpersonal demands of medical residency. The ttRPG context provided a controlled, low-risk arena in which participants could rehearse and refine these competencies while engaging in fictional yet socially meaningful scenarios. As in other simulation-based learning environments, participants were oriented to the targeted competencies but were not informed about specific narrative challenges in advance, preserving spontaneity and naturalistic interpersonal expression.

Importantly, ttRPGs function as structured social simulations. By adopting fictional characters within a guided narrative, residents engaged in real interpersonal dynamics with psychological distance, facilitating exposure, emotional regulation, and perspective-taking—core CBT mechanisms. The Dungeon Master (DM) intentionally designed narrative conflicts that paralleled clinical realities, such as negotiating under pressure or resolving group disagreements, ensuring that gameplay elicited the same competencies emphasized in traditional SST models. For example, in one scenario, participants could attempt to negotiate a peace agreement between opposing factions—requiring assertiveness, collaboration, and strategic communication—or choose confrontation, in which case the narrative consequences affected subsequent sessions and demanded collective decision-making and conflict management.

The evaluative construct of Social Competence—defined as the integration of thoughts, feelings, and behaviors across short-, medium-, and long-term interpersonal contexts (Del Prette and Del Prette, 1999, 2001, 2005)—served as the conceptual anchor for interpreting participants’ perceived changes in social functioning. Thus, the study design combined (1) a theoretically grounded SST framework, (2) CBT-oriented narrative immersion, and (3) phenomenological examination of lived experience, allowing ttRPG sessions to operate as experiential simulations aimed at strengthening the interpersonal competencies required in residency.

Each weekly session followed a structured five-step format (Otani et al., 2024): (i) rapport-building; (ii) task discussion; (iii) goal setting; (iv) gameplay; and (v) feedback. Gameplay elicited specific interpersonal behaviors, which were immediately followed by guided reflection linking in-game decisions to real clinical challenges. This progression—from simulated practice to reflective integration—constituted the pedagogical mechanism through which gamification supported social skills development and mental health–related insights.

### Data collection

Semi-structured individual interviews were conducted prior to the intervention to assess participants’ perceived social skills, prior role-playing experience, familiarity with the game system, and overall mental health during residency. Each interview was audio-recorded for transcription and analysis. Transcripts were used for qualitative analysis but were not returned to participants.

Focus groups were conducted via video conferencing after the intervention and followed a semi-structured format addressing three domains: (1) experiences prior to the intervention, (2) experiences during the RPG sessions, and (3) perceived impacts on mental health, social skills, and quality of life. All sessions were recorded and transcribed by the research team (Table 2).

**Table 2:** Social interactions among the medical residents.

Relationship between residents before playing RPG	Presence of bond between participants in the pre-RPG group
	Absence of bond between participants in the pre-RPG group
Group interactions on the RPG	Out-of-game relationship projected into the game
	Act on behalf of the group regardless of the consequences
	Apprehension when making decisions for fear of judgment from the group

Three focus groups were conducted across two participant groups. Group 1 (*n* = 6) participated in two sessions, and Group 2 (*n* = 5) in one session, each lasting between one and two hours. Transcriptions were reviewed for accuracy, and participants were given the opportunity to clarify statements. Although formal data saturation was not fully achieved due to the exploratory nature of the study, the material was considered sufficiently rich to support thematic analysis.

Focus groups were conducted separately from gameplay sessions to provide a structured reflective space. Audio and video data from intervention sessions were recorded, and all materials were securely stored on password-protected servers and in restricted-access archives, in accordance with institutional ethics protocols.

### Intervention description

The intervention employed the tabletop role-playing game Dungeons & Dragons (5th edition), a collaborative narrative system in which participants adopt fictional characters and engage in structured social problem-solving. While set in a medieval fantasy context, the pedagogical value lies in its interactive mechanics, which require communication, negotiation, cooperation, conflict management, and decision-making under uncertainty—skills directly transferable to clinical environments.

A custom narrative campaign was developed to mirror interpersonal challenges commonly encountered in residency, such as coordinating treatment decisions, managing disagreements, responding empathically to distress, and navigating emotionally charged interactions. The Dungeon Master (DM) acted as facilitator, presenting dilemmas, portraying non-player characters, and modulating narrative complexity to ensure balanced participation and targeted skill practice (Table 3).

**Table 3:** The experience of playing RPG in the critical skills project.

Preferences among RPG narratives	Preference for Cthulhu as a game system
	Perception of greater adherence in more realistic scenarios
	Preference for Dungeons and Dragons as a game system
	Preference for fantasy scenarios in general
	One-shots to multiply RPG experiences
Preferences among game modes	Preference for face-to-face play
	Challenges with attention and interaction in the online modality
	Online sessions provide a more comfortable environment
	Alternating between online and face-to-face sessions for turn-taking
Character creation	Players create characters they identify with
	Players create characters with characteristics they wish to have
	Players create characters different from themselves
	Players choose classes with the function of being effective in the group
	Players dedicate themselves to creating the character’s backstory
	Players encountered difficulties in character creation
	Excessive projection aids in the development of Social Skills
	Increased projection aids in the development of Social Skills
	Both projected and non-projected characters can offer benefits
Character interpretation	Using race and class as an in-game challenge
	Players develop the character’s personality throughout the campaign
	Even with a character’s backstory, the player’s projection remains evident.
	Conflicts between player and their character in roleplaying and decision making
	Difficulties with orientation and character personality traits
	Difficulties in roleplaying with characteristics divergent from their own
	Struggles in expressing the character’s backstory within the campaign
	Difficulties with characters of a different gender than the player
	Self-imposed pressure to win the game
RPG analysis and criticism	Difficulties with the digital tools used
	Disliking the long session periods
	Difficulties in immersion due to not understanding the fantasy world
	Challenges stemming fromlack of knowledge on game mechanics
	Players not taking responsibility for the fictional scenario
	Dungeon Master needs to be more active in providing challenging situations
	Self-assessment form should have a free space for participants to write

Through character embodiment and collaborative storytelling, participants rehearsed interpersonal behaviors within a psychologically safe context. This structure ensured that the RPG sessions functioned not as entertainment alone but as intentional experiential simulations directly linked to the social and emotional competencies required in medical training.

### Data analysis

The qualitative data underwent an inductive content analysis process to identify emerging themes from each developmental phase of the intervention. JVCG and HAD independently and iteratively analyzed the transcripts, validating their findings through mutual consensus. The themes, categories, codes, and excerpts from participant narratives were managed using Miro, an online whiteboard platform. Access to the software was restricted to the researchers throughout the data analysis process. Themes were derived from the manuscript data (Table 4).

**Table 4:** Mental health impacts of role-playing games (RPGs).

Impacts on social skills and emotional development	RPG facilitated reflexivity
	RPG exercised assertiveness in social skills
	RPG exercised communication skills
	RPG provided safe space for new behaviors in different social situations
	RPG exercised planning and decision making
Impacts on group relationships, coordination and cooperation	RPG fostered integration and bonding within the group
	RPG instilled confidence in the group
	RPG developed teamwork
	RPG helped establish roles among the resident group
	RPG was a space for care and social interaction during the pandemic
Resident’s perceptions of clinical interventions with RPG	Recommendations for children and youth
	Recommendations for anxious or phobic patients
	Caution when working with schizophrenic or autistic patients using RPG

## Results

From the content analysis conducted, the findings were systematically organized into themes, categories, and codes. Four main themes emerged from the participants’ responses: Individual Experiences of the Medical Residents During Isolation, Social Interactions Among the Medical Residents, The Experience of Playing RPG in the Critical Skills Project, and Impacts on Mental Health by Playing RPG.

### Individual experiences of the medical residents during isolation

The isolation brought by the COVID-19 pandemic exacerbated the already intense pressures faced by medical residents, who, while learning to navigate their professional responsibilities, were also experiencing significant emotional and psychological strain. The residents found themselves confronting long hours, difficult patient cases, and limited social interactions, all of which were compounded by the isolation protocols. Many residents struggled with balancing the professional demands of residency with their mental well-being, leading to an increase in emotional and behavioral challenges. This theme captures their individual experiences during this unique and difficult time.

#### Stress related residency experiences

Despite recognizing the value in the activities proposed during their residency, participants noted a significant escalation in stress levels as they progressed through the various stages and years of residency. This increase in stress was closely linked to the growing demands of their work, which included a higher volume of patients and more complex clinical cases. These challenges were particularly evident in their experiences at CAISM (Center for Integrated Attention to Mental Health), a specialized mental health service that provides care to patients with severe psychiatric disorders. At CAISM, residents faced the dual pressures of managing an increased patient load while dealing with the intricate nature of mental health care, which often involves long-term treatments, crisis management, and coordination of multidisciplinary care. One participant shared insights into her emotional state during one of these particularly demanding stages:

*“I’m really stressed out at CAISM* (Center for Integrated Attention to Mental Health), *like really stressed. It’s ruining my life at this stage.” - Resident 5*

#### Emotional and behavioral difficulties

Beyond the stress of managing high patient volumes and complex cases, the COVID-19 pandemic introduced additional emotional and behavioral challenges. The isolation measures imposed during the pandemic amplified existing difficulties, such as shyness, anxiety, and a lack of assertiveness. The inability to interact with colleagues in person heightened feelings of detachment and inadequacy, particularly in developing communication and leadership skills—key competencies for successful medical practice. Residents often found themselves grappling with emotional burdens, feeling underprepared to handle the psychological demands of residency, which were exacerbated by the isolation. This environment further stifled their ability to engage socially and assertively in both their professional and personal lives. One participant shared her personal experience with assertiveness:

*“Outside the game, I struggle to impose my opinion. If I have any doubts, I am not sure what I want to do, it is my difficulty to impose what I think is right, what I think I want.” - Resident 2*

Participants also reflected on how they applied insights from the RPG sessions to recognize and manage their anxiety symptoms. Through role-playing, some residents found a therapeutic release, allowing them to explore alternative behaviors in a safe environment that encouraged emotional flexibility and creativity. One participant described how embodying her character in the game led to greater courage and emotional resilience in real life:

*“It would be more courageous, because I'm very worried, anxious, afraid of things going wrong, I do everything righter, and then with the character, this is not necessary, the world goes on without you being like this. It gave me more courage” - Resident 1*

#### Challenges with imagination in a fantasy-based RPG world

While some participants embraced the fantasy elements of the RPG sessions, others struggled with the imaginative aspects of the game. For residents accustomed to structured, real-world medical environments, the abstract and symbolic nature of RPGs posed a significant challenge. The need to imagine fantastical settings and scenarios, far removed from their everyday experiences, created mental blocks for some participants. This difficulty in immersing themselves in the game’s creative narrative limited their ability to engage fully with the scenarios designed to improve their social skills. One participant elaborated on the struggle to connect with the fantasy world:

*“I have a huge mental block when it comes to imagining things, to the point that I struggled a lot with the scenario we created for the project, which was entirely unrealistic and completely unfamiliar to me.” - Resident 9*

On the other hand, two participants reported difficulty in generalizing the behaviors learned within the RPG to real-life situations. They acknowledged the value of the game for experimenting with new behaviors, but felt that the fictional nature of the scenarios created a barrier to applying these lessons outside the game. One resident expressed frustration over this limitation:

“*The biggest difficulty I have to incorporate the gains I feel with Radja (participant’s character during the project) in my real life is because I know that the situations are unreal, they are all parts of a game.” - Resident 7*

#### Internet use to build friendships

The isolation caused by the pandemic significantly limited residents’ opportunities to build or maintain friendships in the traditional ways. Some residents expressed that they had never used the internet to establish friendships before the pandemic and were uncomfortable with this method. Others found the online format to be a viable alternative, especially within the structured environment of RPGs. For those who had previously participated in online role-playing communities, engaging with others in a virtual setting was a more familiar and comfortable way to form meaningful relationships. One participant, who had prior experience playing RPGs online, shared her reflections:

*“I used to play roleplaying games, but even when I played RPG on a forum that was just storytelling, I made some very lasting friendships. I don’t talk to these people anymore because it’s been many years, but those were very good friendships.” - Resident 5*

#### Motivation for playing RPG

When asked about their motivation for participating in the RPG intervention, participants provided a range of reasons. For some, the opportunity to play an RPG was a new and intriguing experience—something they had been curious about but had never had the chance to try before. Others saw the RPG sessions primarily as part of the research project and were motivated more by the academic aspect than the game itself. One resident shared how curiosity drove their involvement:

*“I had always been very curious to play, but I had never had the opportunity.” - Resident 3*

For other participants, particularly those who had never engaged with RPGs or games in general, the social isolation caused by the pandemic was a major factor. The chance to engage in a collaborative, interactive experience helped to alleviate the boredom and loneliness of quarantine. As one participant mentioned:

*“I’ve never been into playing games, I’ve never been into video games. And I participated more because of the… Honestly, for being totally bored at home during this pandemic thing.” - Resident 4*

### Social interaction among the medical residents

#### Relationship between residents before playing RPG

Before engaging in the RPG sessions, participants reflected on the limited social interactions they had with their peers. Medical residency is often an isolating experience, where long hours, demanding responsibilities, and individual rotations minimize the opportunities for residents to build strong, meaningful relationships. This lack of connection was exacerbated by the pandemic, which imposed additional barriers to socialization. While some residents mentioned pre-existing relationships with colleagues from previous academic experiences or shared rotations, the majority had little to no substantial interactions with each other outside of their professional responsibilities. This environment created a social void, which RPG sessions later helped to fill. One participant explained their sense of isolation:

*“I think I was the one who knew people the least because I hadn’t studied with them. I’m not in anyone’s group.” - Resident 1*

#### Group interactions on the RPG

The RPG format introduced unique dynamics into group interactions. From the onset, one of the key rules in the RPG sessions was that participants’ characters should not know each other within the fictional world. This setup mirrored the reality of medical residency, where new team members must quickly develop rapport and trust without prior connections. The RPG allowed residents to simulate building friendships and communication skills in a controlled, yet immersive, setting. However, despite the fictional context, some participants found it difficult to separate their in-game relationships from their real-life bonds. They noted that their established real-world dynamics often influenced their decisions within the game, sometimes prioritizing group harmony over individual growth. One resident observed this phenomenon:

*“I didn’t feel that we built this friendship in the story. I felt that we were never able to separate the friendship from real life.” - Resident 9*

The focus on group cohesion sometimes had unintended consequences. Some participants expressed hesitation in making bold or risky decisions in the game, out of fear of being judged by their peers. This mirrored their real-life experiences of being cautious in social interactions within the stressful environment of residency. One participant described the internal conflict between playing authentically and managing group perception:

*“I was very stuck, like, ‘Oh, what is the group going to think? What will the group think if I act too crazy here now?’ which I think is a trait of mine that I couldn’t get rid of during the game.” - Resident 10*

### The experience of playing RPG in the critical skills project

#### Preference among RPG narratives

As residents gained more exposure to RPGs, they began to express preferences for certain types of narrative styles. Some participants gravitated toward systems like “The Call of Cthulhu,” which blends horror and reality in a way that feels more grounded and accessible for those unfamiliar with fantasy genres. This preference was rooted in their comfort with real-world analogs, as the storylines in these games typically mirrored familiar moral and ethical dilemmas encountered in their medical practice. For these participants, narratives closer to reality were more immersive because they required less imagination and allowed for deeper emotional and intellectual engagement. One resident shared:

*“I was just thinking that for the general public… maybe Cthulhu would fit better, because it doesn’t require so much immersion and doesn’t have a complex fantasy.” - Resident 3*

Conversely, others favored the high-fantasy setting of Dungeons and Dragons, appreciating the freedom and creativity that such worlds offered. For them, the fantasy elements created a safe space where they could explore new behaviors and attitudes without the weight of real-world consequences. The fantasy context allowed residents to experiment with assertiveness, problem-solving, and empathy in a way that felt removed from the constraints of their professional lives. One participant explained:

*“I felt that in the fantasy world I had a lot more options to… to ‘trip’ and to be much more protected from the consequences of my actions.” - Resident 6*

Participants also suggested that incorporating more one-shots—shorter, self-contained RPG sessions—could diversify the experiences within the project. These condensed campaigns allowed players to explore different systems, characters, and scenarios, which they found valuable for skill development. One resident expressed this view:

*“We learn more about RPG, you know? Because otherwise we get too stuck to a single story and keep seeing only that. And I think that for people who had no contact with RPGs, seeing other games, characters and systems was very productive.” - Resident 9*

#### Preferences among game modes

The choice between face-to-face, hybrid, or online RPG sessions also revealed diverse preferences among the residents. A significant number of participants preferred face-to-face interactions, citing the importance of non-verbal communication and direct eye contact in enhancing the gaming experience. They felt that physical presence allowed for a deeper connection, not only with the game but also with each other. This interaction helped them to better interpret social cues like voice tone, facial expressions, and body language, which are all crucial for effective communication and collaboration in their medical careers. One participant noted:

*“I prefer it face-to-face because I think there’s a part that we lose from playing online, which is a character interpretation thing, eye to eye and reaction.” - Resident 5*

However, some residents appreciated the online format for its convenience, particularly during the pandemic. Online sessions allowed them to engage with the game from the comfort of their homes, providing a sense of security and protection from the vulnerability of in-person interactions. For residents who were less comfortable with the social aspects of RPGs, the distance provided by online sessions reduced social anxiety. One resident shared their feelings on this aspect:

*“Personally, that would be even more challenging for me. In the online sessions, although it’s still a little difficult for me, but I feel a little more… protected.” - Resident 7*

In light of these differing preferences, one participant suggested a hybrid model, which would offer the best of both worlds—combining the benefits of face-to-face and online formats:

*“Maybe I could even do some face-to-face sessions and some online sessions next time, you know? Because I think both have a positive sides.” - Resident 8*

#### Character creation

The process of creating a character in RPGs provided residents with an opportunity to project aspects of their own personalities or explore traits they desired to develop. Many participants described how their characters were mirrors of their real selves, embodying their psychological, emotional, and even physical attributes. For some, this projection allowed them to confront and address personal challenges in a fictional space. One resident reflected on how their character resembled them:

*“I created a character totally projecting myself. It was who I am, expansive, inappropriate and sometimes unhappy in most of its comments, but I was trying, as time went on, to use the session to work out some things I do that bothers other people on a day-to-day basis.” - Resident 6*

Other residents chose to create characters that embodied traits they wished to develop, such as assertiveness, or even characters with personalities entirely opposite to their own. This creative freedom allowed participants to practice new behaviors in a controlled and supportive environment, helping them gain confidence and social skills. One resident highlighted this strategy:

*“Imposing limits, which is something I have a little difficulty doing in my daily life. And then being in the role of Nina made me a lot more comfortable doing that.” - Resident 8*

A decisive factor for some players was to consider the potential needs of the group when crafting their characters. They recognized the importance of group diversity in character creation, striving to ensure a balance of classes, races, and roles within the team. This approach reflects how, in real life, residents must collaborate with colleagues who bring different skill sets to the medical team. They were conscious of contributing unique strengths to the group dynamic, which paralleled the importance of role specialization in their professional environments. One resident described this strategic approach to character selection:

*“I wanted to get a class, a race that was different from the others, because at the RPG tables I’d played before, having different characters, races and classes, was always cool, it was always something that brought a lot of variety to the game and many advantages in many situations. So, I kind of waited for the crowd to choose and I picked last because I wanted something that was different and could contribute to the group.” - Resident 2*

Residents expressed that character creation extended beyond technical skills, shaping their characters’ values, motivations, and moral stances. This reflection on personal values allowed them to explore the tension between personal and professional identity within a fictional framework. The process of building a character’s backstory offered a way to solidify these aspects and better understand their role within the game, an exercise that mirrored the introspection often required in clinical practice. One resident shared the significance of this narrative work:

*“Writing the story helps me a lot in building the character’s personality according to the decisions he made in life, the things that happened.” - Resident 2*

However, the wide array of character options—from races and classes to moral orientations—also added layers of complexity to the process. For beginners, this richness could be overwhelming, as they struggled to navigate the many choices without prior knowledge of the game mechanics. This challenge mirrors the experience of medical residents who are confronted with a complex, fast-paced environment where they must quickly master new skills. One resident described their difficulty with character creation:

*“At first, I was trying to create the character thinking of a story, only making the more technical decisions of skills, class and race, secondarily, and I was finding it more difficult to adapt because I didn’t really know the rules and possibilities of how to execute the ideas.” - Resident 3*

While projecting personal characteristics onto their characters offered a form of self-expression, participants also debated whether this strategy hindered or helped their development. One participant reflected on the potential downside of creating a character too similar to themselves, which limited their opportunities to challenge personal growth through the game:

*“I infused so many of my own characteristics into my character that I had little incentive to try to develop skills I struggle with.” - Resident 9*

On the other hand, some participants felt that having a character similar to oneself allowed for a more direct and honest simulation of personal difficulties, making the training more impactful:

*“On this issue of developing social skills, which is the project’s objective, I think the more you can put yourself into the character the better you will be at evaluating this whole issue.” - Resident 7*

Ultimately, a third resident suggested that both approaches—creating characters similar to or different from oneself—offered valuable learning opportunities:

*“I don’t think it needs to be such a rigid thing, like, ‘you have to create a character that resembles you’ or ‘that’s completely opposite to you,’ I think you can work on both fronts.” - Resident 6*

#### Character interpretation

During the sessions, participants highlighted the ways in which they used their characters to challenge themselves by experimenting with new roles and identities. For more experienced players, selecting different races and classes provided a way to push the boundaries of their usual habits and explore different ways of interacting within the game. This experimentation reflects the process of exploring new behaviors in clinical settings, where medical residents often try different communication styles or strategies to improve their interactions with patients and colleagues. One participant remarked on the novelty of this approach:

*“One thing that brought me closer was that I took a class I’d never played before and a race I’d never played before. So, I am discovering new things on how to readjust some old habits I had in relation to the game itself, and it’s been very interesting, it’s been new for me. It’s like rediscovering the game, because these are decisions I wouldn’t have made before.” - Resident 2*

In addition to these technical choices, the evolution of characters over time became an important aspect of gameplay for the residents. As the sessions progressed, participants used their characters as tools to explore new attitudes, emotions, and ways of interacting with others, highlighting the developmental aspect of RPGs. One resident described the transformation of their character:

*“I think Nina was a little intimidating, a little aggressive, and at a certain point in the game I said… I kind of started to make myself vulnerable.” - Resident 8*

However, participants also noted the moral and emotional complexities that emerged during gameplay, particularly when their characters’ actions conflicted with their own personal values. This tension highlighted the ethical dilemmas they often face in medical practice, where decisions can sometimes conflict with personal beliefs. One participant shared their struggle with this overlap:

*“I never play with evil characters because I can’t interact with other characters having an evil character. For me, the fun of RPG lies precisely in the interaction between the characters, so it’s very difficult for me to play an evil character.” - Resident 2*

Some residents also experienced challenges in fully expressing the backstories and motivations they had developed for their characters within the game’s narrative structure. These frustrations mirrored the limitations they sometimes faced in real-world clinical settings, where the pressure of the moment often leaves little room for in-depth exploration of patient history. One resident commented on this difficulty:

*“I couldn’t put everything I had thought about his background, interests, motivations, with the course the story was taking.” - Resident 7*

For one male resident, playing a female character introduced an unexpected challenge. He found it difficult to portray the nuances of the opposite gender, despite initially assuming the gender switch would not be a significant barrier. This experience led him to reflect on how different social roles can be difficult to inhabit, particularly when one lacks lived experience. He explained:

*“I… it’s just that, there aren’t many characteristics of my character that are typical of the female gender, but maybe I found it difficult interpreting and being in the body of a woman with the same difficulty I feel interpreting and being in the body of an elf that I’ve never had an earthly experience to know what it’s like.” - Resident 3*

Residents commented that the experience of playing RPG was influenced by their familiarity with video games, where the primary objective is often to “win” rather than engage in cooperative problem-solving or narrative exploration. This mindset, ingrained from years of video game exposure, led some participants to approach the RPG sessions with a competitive mentality, rather than focusing on social dynamics or interpersonal growth. One participant expressed this challenge, revealing how it shaped his behavior during the game:

*“It was as if I approached the game more like a video game where you have to win, you know? So, it was like, ‘What do I need to do to win the game?” - Resident 11*

#### RPG analysis and criticism

Throughout the focus groups, participants provided constructive criticism of the project, pointing out technical and procedural difficulties that impacted their engagement. One key issue was the limitations of the online tools used for character management and video conferencing. These platforms often created barriers to seamless interaction, especially when it came to the spontaneity required for role-playing. One resident described how these technological hurdles affected their ability to fully engage with the group:

*“Sometimes I felt that online, here, through the video conference, sometimes I wanted to interact and didn’t have the timing of the thing, because you know… we’re talking one at a time, and you can’t figure who wants to talk. So, sometimes we stopped interacting with something or we only managed to do it if you were able to interrupt talking at a very specific moment and I think that left a little to be desired in terms of the resourcefulness of the thing itself.” - Resident 3*

Another common criticism focused on the duration of the RPG sessions, which were considered too long, particularly for participants who were new to the format. The extended length of the sessions made it difficult for some residents to maintain focus and enthusiasm, especially those who were unfamiliar with RPGs. One participant commented on the excessive length:

*“I sometimes feel that the duration is a bit lengthy, especially for those who have never played before. I think four hours is a little too much.” - Resident 1*

For some residents, grappling with the fantastical elements of RPG presented a significant challenge. The imaginative demands of the game, where players must immerse themselves in a fictional world, were difficult for those unaccustomed to playful or imaginative activities. This barrier was particularly prominent among residents who did not regularly engage in games or other creative pursuits. One participant explained the difficulty they faced in adapting to the fantasy world:

*“I’m not much of a gamer, so playful activities are something I don’t have much experience with. It’s not part of my routine or something I’ve done in my life. And then it’s hard for me to let go and do the role playing myself.” - Resident 1*

In addition to these challenges, limited understanding of the game mechanics hindered the experience for some participants. Without prior knowledge of the rules, spells, and strategies of the RPG system, players found themselves struggling to make decisions within the game. This lack of familiarity with the system paralleled the early stages of their medical training, where mastering the technical aspects of clinical practice was a daunting task. One participant shared their frustration:

*“I think it made it difficult for me even in a matter of ‘oh… which spell should I cast?’ You know, I had zero knowledge… and barely knew the spells I had.” - Resident 8.*

Several players also commented that the fictional world of RPG did not have a significant impact on their real-world attitudes. They noted that certain social dynamics and interpersonal situations could have been more pronounced if the Dungeon Master (DM) had taken a more active role in presenting them. One participant openly suggested that the DM could have better tailored the scenarios to address specific social skills or difficulties that residents were facing:

*“Perhaps the DM could have pushed a little bit or even when someone mentions that it in the feedback, the DM should say ‘well, if this person has difficulties with this and wants to work on it, maybe I could incorporate more situations of this type.’” - Resident 6.*

Residents also proposed a revision of the feedback and self-assessment forms, suggesting that these forms should include more open-ended sections where participants could express personal thoughts that may not fit the structured questions. One participant emphasized the value of having additional space for reflection:

*“Sometimes I wanted to say some things, but if I said them here, I think they wouldn’t have much to do with what was being asked and I don’t know if it would make sense to talk about them. So, sometimes if it was a form that gave me some more space…” - Resident 7*

### Impacts on mental health by playing RPG

#### Impacts on social skills and emotional development

Residents frequently discussed the positive effects of RPG sessions on their social skills and emotional well-being. The immersive nature of role-playing allowed them to practice assertiveness, empathy, and reflexivity in a safe and supportive environment. These skills are critical in both personal and professional contexts, and participants noted how the game gave them an opportunity to develop these qualities in a non-threatening setting. One player reflected on how therapeutic the experience had been:

*“It’s kind of therapeutic for me in some ways, I’m able to realize some things about my behavior.” - Resident 5*

In particular, residents appreciated how RPGs provided a low-stakes environment where they could experiment with different actions and decisions without fear of real-world consequences. This sense of safety and lack of social judgment enabled them to take risks, make mistakes, and reflect on their choices. One participant shared how this aspect of RPGs shifted their mindset:

*“I believe one of the great aspects of RPGs is that they allow us to find a certain peace with our decisions and recognize that sometimes, things are beyond our control. Whether things go wrong or right, it’s a collective experience, and that’s what makes it so special.” - Resident 6.*

#### Impacts on group relationships, coordination and cooperation

RPGs also fostered stronger group cohesion and teamwork among the residents. The collaborative nature of the game helped them to build rapport, particularly with peers they had not interacted with closely before. Through the game, participants developed a better understanding of each other’s strengths and how to work together more effectively, a skill directly transferable to their medical training. One participant described the importance of the bonds formed through RPG:

*“I think the first thing is the bond between us, we play among us who are residents. I think this strengthens our group as a whole, you know? We get to know each other better and maybe we can work better with each other because of this bond.” - Resident 5*

The non-hierarchical structure of the RPG sessions also allowed leadership to emerge organically within the group, based on the context of the game and the specific abilities of each character. This dynamic paralleled the interdisciplinary teamwork often seen in medical settings, where leadership roles shift based on the situation at hand. One resident shared their thoughts on this aspect of teamwork:

*“I believe it boils down to our ability to work within a group, acknowledging that there are other professionals and team members with roles different from ours, and respecting those roles as well.” - Resident 5*

Participants also noted that RPGs played a critical role during the pandemic by providing a much-needed social outlet. The enforced isolation of COVID-19 had limited their social interactions, and the game became a valuable tool for maintaining social connections and reducing loneliness. One participant described how RPGs helped him navigate this period of social distancing:

*“Yeah, it’s the way to socialize, you know? To have a minimum of interaction outside your house. I think it’s been very good for me in this sense, to exchange experiences at a time when everyone is at home, reclusive…” - Resident 4*

#### Residents’ perceptions of clinical interventions with RPG

Participants expressed a consensus that the playful and imaginative nature of RPGs makes them particularly well-suited for use with children and adolescents, a demographic often resistant to more traditional forms of psychological treatment. Teenagers, in particular, were seen as a group that could benefit from the immersive and interactive qualities of RPGs. One resident reflected on how impactful RPG sessions might have been during their own teenage years:

*“If I were a teenager doing this… it would have been really revealing. So… the way of dealing with people, of having greater security, of having… of having a place to speak, you know? There for you…” - Resident 11*

This sentiment highlights how role-playing games could be used as a tool to help teenagers develop social skills, gain self-confidence, and navigate complex interpersonal situations in a safe, structured environment. The flexibility of RPGs, where players can take on different roles and explore diverse scenarios, was viewed as an advantage in engaging adolescents who might otherwise shy away from therapy.

Participants also noted the potential of RPGs for therapeutic interventions targeting patients who struggle with social anxiety or other interaction-related disorders. The game’s inherent focus on collaboration and communication could provide a lower-risk environment where patients could practice and improve their social skills without the immediate pressures of real-world interactions. One participant discussed how this approach might be beneficial for socially anxious patients:

*“Wow, the idea of social phobic patients came to my mind a lot, you know? To take a patient to an interaction with less risk, but still requiring them to deal with other human beings, but in a less abrupt exposure, too.” - Resident 2*

By offering a gradual introduction to social interaction, RPGs could bridge the gap between isolation and social participation. The symbolic distance between the game world and real life may help patients feel less vulnerable, allowing them to experiment with social behaviors that they might find overwhelming in reality.

However, residents also recognized the need for caution and adaptation when applying RPGs to clinical settings, especially with patients who have cognitive or psychotic disorders such as schizophrenia or autism. RPGs demand a certain level of abstraction and projection, which could be challenging for individuals whose ability to separate fantasy from reality is compromised. One resident, in particular, raised concerns about how individuals with schizophrenia might become overly immersed in the game, blurring the line between their character and themselves:

*“Be careful with schizophrenics, okay?! One could go psychotic and start to think he’s the real character.” - Resident 2*

This concern underscores the necessity of careful monitoring and tailored interventions when using RPGs in therapeutic settings, particularly for vulnerable populations. The participants suggested that, while RPGs offer a valuable and flexible tool for therapy, the nature of the game and the specific needs of the patient should always be taken into account to avoid exacerbating symptoms.

## Discussion

This study aimed to assess the feasibility of using a role-playing game (RPG) for Social Skills Training among medical residents. The qualitative analysis revealed perceived positive impacts on group cohesion, communication, cooperation, assertiveness, empathy, and broader aspects of mental health and quality of life during the SARS-CoV-2 quarantine period. These findings suggest that RPG-based interventions may represent a novel and promising approach within medical education. Although RPGs have demonstrated psychological and prosocial benefits in other educational settings, no prior studies have specifically examined their use as a structured social skills training tool for medical residents.

The mechanisms underlying these outcomes appear to be linked to the structured yet flexible nature of RPG-based social simulation. Through engagement with narrative dilemmas requiring negotiation, emotional regulation, collaboration, and perspective-taking, residents rehearsed interpersonal behaviors closely aligned with real clinical challenges, such as coordinating care decisions, managing disagreements, and responding empathically under pressure. The fictional context and narrative distance reduced performance anxiety and evaluative threat, creating a psychologically safe space for experimentation and reflection.

Unlike traditional simulation models in medical education—often centered on technical competencies or scripted patient interactions—RPG-based gamification promotes spontaneous dialogue, collaborative storytelling, and real-time narrative modulation. This format allows complex interpersonal dynamics to emerge organically while remaining guided by the facilitator. In this study, the Dungeon Master (DM) intentionally structured dilemmas that elicited assertiveness, conflict management, leadership, and cooperative problem-solving, paralleling competencies essential in residency. The combination of narrative immersion and structured reflection appears to function as an experiential learning process consistent with cognitive-behavioral and social learning principles, whereby repeated practice and feedback support skill generalization beyond the simulated environment.

Participants also emphasized the relevance of the intervention during the pandemic context. The enforced social isolation amplified existing stressors associated with residency, including emotional exhaustion, uncertainty, and performance pressure. Within this context, the RPG sessions were perceived not only as skill-building exercises but also as opportunities for social connection and emotional expression. Residents reported increased bonding, improved group coordination, and a sense of shared experience that extended beyond gameplay. These findings are consistent with prior literature highlighting the mental health vulnerabilities of medical trainees and the protective role of social skills and peer support during periods of high occupational stress.

It is important to clarify that this study was not designed to measure causal or pre–post changes in social skills or mental health. As a phenomenological and exploratory investigation, the focus was on participants’ subjective experiences and perceived interpersonal development. The reported improvements reflect perceived gains rather than objectively measured outcomes. Future research should incorporate validated quantitative instruments administered at baseline and post-intervention to evaluate measurable effects and further test the feasibility suggested by these qualitative findings.

The findings align with broader literature indicating that RPG participation can foster emotional regulation, reflexivity, and social connectedness in educational contexts. The intervention’s adaptability—online, face-to-face, or hybrid—combined with minimal material requirements, suggests practical feasibility for implementation in diverse training environments.

This study has limitations. The sample was small, non-random, and limited to psychiatry residents from a single institution. The absence of a control group restricts causal interpretation. However, given the exploratory nature of this initial investigation, the primary objective was to assess experiential feasibility and identify potential facilitators and barriers to implementation. Participant feedback informed iterative refinement of the intervention and highlighted practical considerations for future scaling.

The data generated also informed preliminary implementation insights guided by the Consolidated Framework for Implementation Research (CFIR). CFIR provides a structured approach to identifying contextual and organizational factors that influence intervention adoption and sustainability (Damschroder et al., 2009). Mapping emergent themes onto CFIR domains may support future multi-level implementation studies of RPG-based social skills interventions in medical education.

## Conclusion and future directions

This exploratory study evaluated the feasibility of Social Skills Training through RPG during the SARS-CoV-2 pandemic using qualitative discourse analysis. While further investigation incorporating quantitative pre–post assessments is warranted, participants reported perceived improvements in interpersonal relationships, communication, cooperation, assertiveness, empathy, and aspects of mental health and quality of life. These findings suggest that structured RPG-based interventions may represent a complementary and innovative approach for fostering social and emotional competencies in medical residency training.

## Data Availability

The raw data supporting the conclusions of this article will be made available by the authors, without undue reservation.
